# Multiple transactivation domains of EZH2 bind to the TAZ2 domain of p300 and stimulate the acetyltransferase function of p300

**DOI:** 10.1042/BCJ20253037

**Published:** 2025-07-02

**Authors:** Dustin C. Becht, Soumi Biswas, Chenxi Xu, Hongwen Xuan, Moustafa Khalil, Ling Cai, Catherine A. Musselman, Xin Liu, El Bachir Affar, Xiaobing Shi, Gang Greg Wang, Tatiana G. Kutateladze

**Affiliations:** 1 Department of Pharmacology, University of Colorado School of Medicine, Aurora CO 80045, U.S.A.; 2 Department of Pharmacology and Cancer Biology, Duke University School of Medicine, Durham NC 27710, U.S.A.; 3 Department of Pathology, Duke University School of Medicine, Durham NC 27710, U.S.A.; 4 Department of Epigenetics, Van Andel Institute, Grand Rapids, MI, U.S.A.; 5 Maisonneuve-Rosemont Hospital Research Center, Montréal, Québec H1T 2M4, Canada; 6 Department of Biochemistry and Molecular Genetics, University of Colorado School of Medicine, Aurora CO 80045, U.S.A.; 7 Cecil H. and Ida Green Center for Reproductive Biology Sciences, The University of Texas Southwestern Medical Center, Dallas TX 75390, U.S.A.; 8 Department of Medicine, University of Montréal, Montréal, Québec H3C 3J7, Canada

**Keywords:** chromatin, EZH2, p300, TAZ2, transactivation domain

## Abstract

The H3K27me-specific methyltransferase enhancer of zeste homologue 2 (EZH2) is the catalytic subunit of the repressive complex *Polycomb* repressive complex 2. EZH2 is typically implicated in transcriptional silencing, but it can also activate gene expression. Here, we show that EZH2 contains three adjacent transactivation domains (EZH2_TAD_) that are recognized by the TAZ2 domain of the transcriptional coactivator and acetyltransferase p300 (p300_TAZ2_). Binding interfaces identified by chemical shift perturbations in NMR experiments, measurements of binding affinities, and analysis of the complex formation by mass photometry demonstrate that each EZH2_TAD_ can be concomitantly bound by a separate p300_TAZ2_. Interaction of EZH2_TAD_s with p300_TAZ2_ stimulates H3K18- and H3K27-specific acetyltransferase activity of p300. We show that in 22Rv1 prostate cancer cells, EZH2 occupies a large set of gene loci lacking H3K27me3, and these non-canonical genomic sites are instead co-occupied by p300, RNA Polymerase II and BRD4 and are rich in histone marks associated with transcriptional activation. Our findings shed light on the potential basis for such a high degree of genetic co-localization through the direct association of p300_TAZ2_ with EZH2_TAD_s.

## Introduction

Enhancer of zeste homologue 2 (EZH2) is the catalytic subunit of *Polycomb* repressive complex 2 (PRC2), a major transcriptional regulator that restricts lineage-specific gene activation and maintains cell identity [1,2]. The PRC2 complex methylates lysine 27 of histone H3, producing the epigenetic mark H3K27me3, a hallmark of gene repression [1,3-5]. EZH2 and the scaffolding subunits SUZ12 and EED form a minimal assembly that retains methyltransferase activity and, together with another subunit RBBP7/4, comprise the PRC2 core [6]. In addition, several non-core accessory proteins, such as AEBP2, JARID2 and PCL1-3, can associate with this core, leading to the formation of distinct subcomplexes [7-12]. Recruitment of PRC2 to the particular genomic loci and its release require multiple co-operative and competitive contacts with DNA and histones, often involving several subunits of the complex [13,14].

The methyltransferase activity of the su(var)3–9, enhancer of zeste, trithorax (SET) domain of EZH2 can be allosterically regulated intramolecularly through α-helical motifs, or modulated intermolecularly by other PRC2 subunits, and pre-existing H3K27me3 [7-10]. Generally, EZH2 is viewed as a transcriptional silencer; however, recent studies have linked it to PRC2-independent gene activation. EZH2 stimulates gene transcription in cancer cells and has been found to be overexpressed in a wide array of cancers [15-18]. Although much less is known regarding this non-canonical PRC2-independent function of EZH2, biochemical studies identified a series of partially disordered helices of EZH2 that can serve as transactivation domains (TADs) to recruit transcriptional activators, such as c-Myc and p300 [19-21].

p300 and its paralogue CREB-binding protein (CBP) are histone H3K18- and H3K27-specific acetyltransferases and transcriptional coactivators [22-26]. p300 contains a diverse set of protein- and DNA-binding modules that interact with activators and basal transcription factors forming multi-subunit complexes required for cell proliferation, differentiation, apoptosis and many other normal cellular processes [27-30]. p300 enzymatic activity is regulated through the autoinhibitory loop of the catalytic histone acetyltransferase (HAT) domain, which, in a hypoacetylated form, suppresses this activity but releases the inhibition upon hyper-autoacetylation, and through other domains present in p300. These include the acetyllysine binding bromodomain [31-33], the RING-PHD fingers region [34], the ZZ domain that stimulates acetylation of H3K27 and H3K18 *in cis* [35], and the TAZ2 domain that associates with transcription factors [36-38].

In the present study, we show that multiple TADs of EZH2 (EZH2_TAD_s) are recognized by the TAZ2 domain of p300 (p300_TAZ2_), and this recognition promotes the enzymatic activity of p300. The increased apparent concentration of the binding sites due to the close proximity of EZH2_TAD_s and multivalent engagement suggest a mechanism for rapid accumulation of p300 at the EZH2 binding sites. This binding mechanism could explain the observed substantial co-localization of p300 and EZH2 at EZH2’s non-canonical genomic targets.

## Results and discussion

### EZH2 contains three TADs for p300_TAZ2_

**Figure 1:**
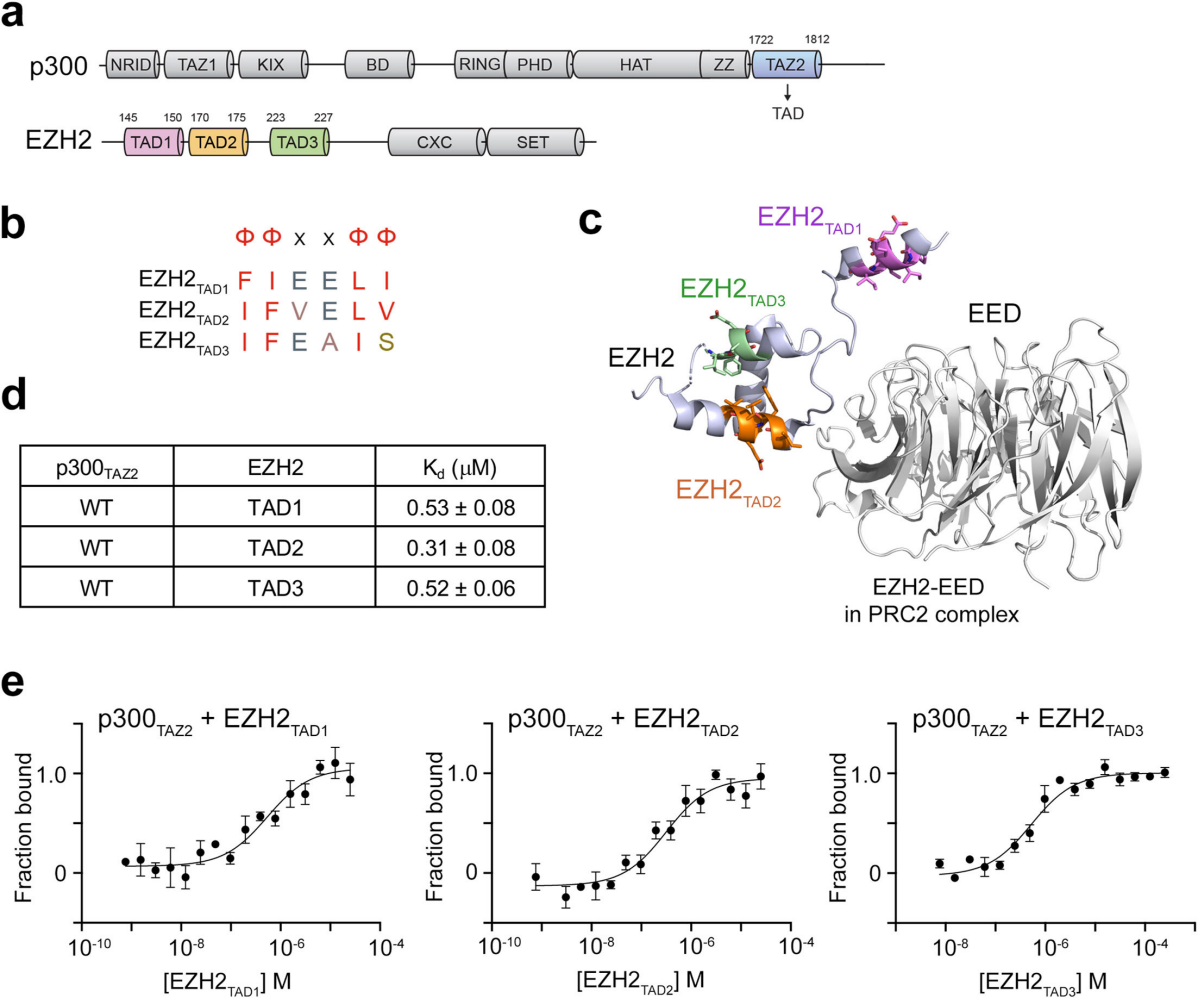
p300_TAZ2_ recognizes three TAD motifs in EZH2. (**a**) Domain architecture for human p300 and EZH2 proteins. (**b**) Sequence alignment of the three TAD motifs found in EZH2, wherein Φ indicates a hydrophobic residue and x indicates any residue. (**c**) A ribbon diagram of the crystal structure of the EZH2 and EED components of the PRC2 complex (PDB: 5HYN). (**d**) Summary of binding affinities determined by MST for the interaction of p300_TAZ2_-His with indicated EZH2 TAD peptides. K_d_ values are the average of three (EZH2_TAD2_ and EZH2_TAD3_) or four (EZH2_TAD1_) independent measurements ± SD. (**e**) Normalized binding curves used to determine K_d_ by MST. Data and point errors are the average of three or four independent measurements ± SEM.

p300_TAZ2_ has been shown to recognize the hydrophobic ΦΦxxΦΦ motif, also known as the TAD, where Φ represents a hydrophobic residue and x represents any residue [39]. EZH2 contains three such motifs, encompassing amino acids 145–150, 170–175 and 223–227 of EZH2 (Figure 1a and b). In the structure of the PRC2 complex [10], the region containing all three motifs is in close proximity to the EED subunit of the complex, although the motifs themselves do not directly interact with EED (Figure 1c). Each motif is found in a separate short α-helix, with the second and third motif-containing α-helices packing against each other and the first motif-containing α-helix positioned away from the other two. To determine whether the EZH2 motifs represent TADs for p300_TAZ2_, we produced p300_TAZ2_ and tested its binding to three EZH2 peptides, each containing a single motif, by microscale thermophoresis (MST). The MST measurements showed that p300_TAZ2_ binds well to either EZH2 peptide, hereafter referred to as EZH2_TAD1_, EZH2_TAD2_ and EZH2_TAD3_ (Figure 1d and e). The interactions of p300_TAZ2_ with the EZH2_TAD_ peptides yielded K_d_s (dissociation constants) of 0.52–0.53 μM for EZH2_TAD1_ and EZH2_TAD3_, respectively, and 0.31 μM for EZH2_TAD2_. These values were in the range of binding affinities observed for the association of p300_TAZ2_ with TADs from other proteins, indicating that the p300_TAZ2_–EZH2_TAD_ interactions are physiologically relevant [36,39,40].

### Mapping the EZH2_TAD_-p300_TAZ2_ binding interfaces

**Figure 2:**
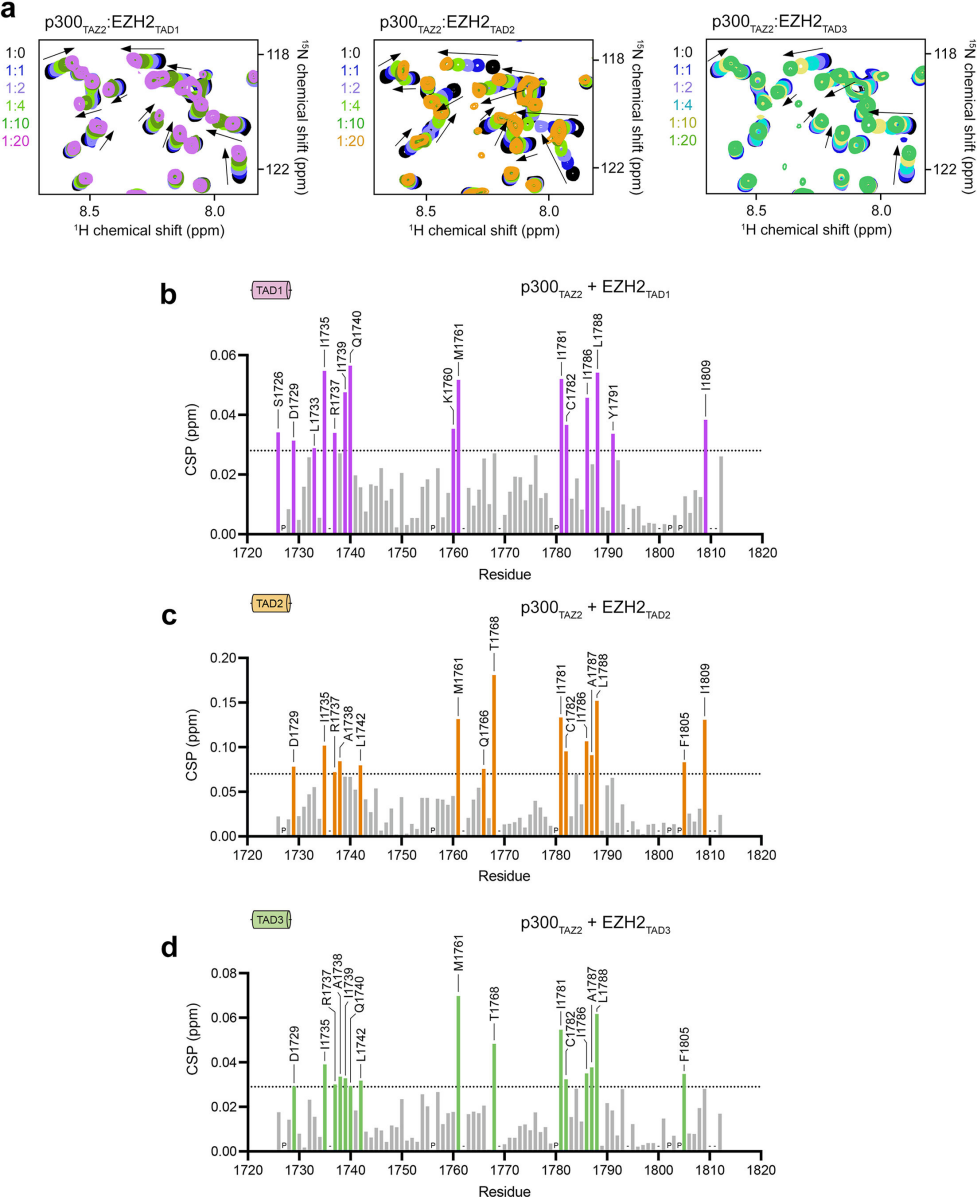
Comparison of CSPs in p300_TAZ2_ upon binding of EZH2_TAD_ . (**a**) Overlaid ^1^H,^15^N HSQC spectra of ^15^N-labeled p300_TAZ2_ collected in the absence (black) and presence of indicated TAD peptides: EZH2_TAD1_ (aa 142–154 of EZH2), EZH2_TAD2_ (aa 167–185 of EZH2), and EZH2_TAD3_ (aa 220–231 of EZH2). Spectra are color-coded according to the protein:peptide molar ratio. See also Supplementary Figure S1. (**b-d**) Normalized CSPs observed in ^1^H,^15^N HSQC spectra of p300_TAZ2_ in the presence of ten molar equivalents of indicated EZH2 TADs. The dotted line defines the perturbations above average plus 0.67 SD, in which a similar number of residues are significantly perturbed by each TAD. Unassigned residues (“–“) and prolines (“p”) are indicated.

To examine the molecular mechanism by which p300_TAZ2_ recognizes three EZH2_TAD_s, we carried out NMR experiments. ^1^H,^15^N heteronuclear single quantum coherence (HSQC) spectra of ^15^N-labeled p300_TAZ2_ were collected, while unlabeled EZH2_TAD_ peptides were titrated into NMR samples (Figure 2a). Addition of each EZH2_TAD_ resulted in chemical shift perturbations (CSPs) in the spectra of p300_TAZ2_, confirming the formation of the complexes between p300_TAZ2_ and either EZH2_TAD1_, EZH2_TAD2_ or EZH2_TAD3_ (Figure 2a). To identify p300_TAZ2_ residues involved in contact with EZH2_TAD_, we reassigned backbone amide resonances of p300_TAZ2_ [37] and plotted CSPs observed in ^1^H,^15^N HSQC spectra of p300_TAZ2_ upon the addition of EZH2_TAD1_, EZH2_TAD2_ and EZH2_TAD3_ per residue (Figure 2b–d). Overall, each EZH2_TAD_ peptide induced CSPs roughly in four regions of p300_TAZ2_, encompassing residues 1730–1740, 1760–1770, 1780–1790 and 1800–1810. Although patterns of CSPs in three NMR experiments were similar, they were not identical. In comparison with EZH2_TAD1_ and EZH2_TAD3_, EZH2_TAD2 _induced CSPs larger in magnitude, and saturation was reached faster, supporting MST data that among the three EZH2_TAD_s, EZH2_TAD2_ binds most tightly. The patterns of CSPs observed upon binding of EZH2_TAD2_ and EZH2_TAD3_ were very similar, whereas CSPs observed upon binding of EZH2_TAD1_ were unique in comparison.

**Figure 3:**
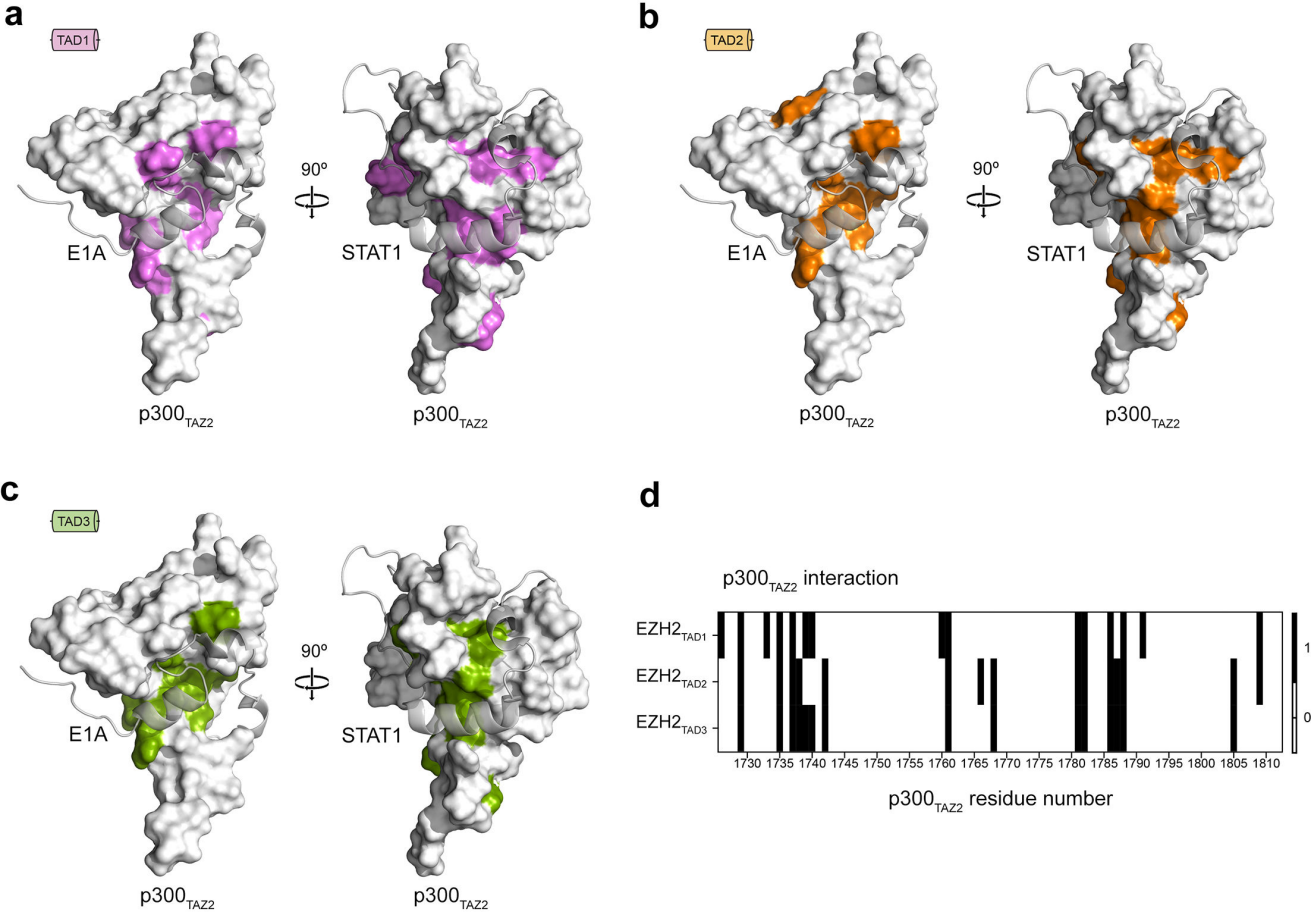
Mapping the EZH2_TAD_ binding sites of p300_TAZ2_ . (**a-c**) CSPs induced by the addition of ten molar equivalents of indicated EZH2_TAD_ peptides mapped onto an NMR structure of p300_TAZ2_ (PDB: 2MH0). The most perturbed residues are colored magenta (a, EZH2_TAD1_), orange (b, EZH2_TAD2_), or green (c, EZH2_TAD3_). E1A (PDB: 2KJE) and STAT1 (PDB: 2KA6) TADs are modeled in the binding pocket of p300_TAZ2_ and depicted in transparent ribbon diagrams. (**d**) Heatmap of the most perturbed (black bars) residues of p300_TAZ2_ upon addition of ten molar equivalents of indicated EZH2_TAD_.

To gain insight into the formation of the complexes, we mapped the most perturbed residues shown in Figure 2b–d onto the structure of p300_TAZ2_ and analyzed all the structures of the p300_TAZ2_-TAD complexes reported to date. We found that CSPs caused by EZH2_TAD_ peptides are well aligned with the binding pockets for E1A or STAT1 peptides in their respective complexes with p300_TAZ2_ (Figure 3a–c). The substantial overlap of the binding sites for EZH2_TAD2_ and EZH2_TAD3_ suggested that these TADs do not concurrently interact with one p300_TAZ2_ domain; however, the more extensive binding site for EZH2_TAD1_ prompted us to investigate whether EZH2_TAD1_ and EZH2_TAD2_ can bind concomitantly (Figure 3d).

### Each EZH2_TAD_ can engage a separate p300_TAZ2_

**Figure 4:**
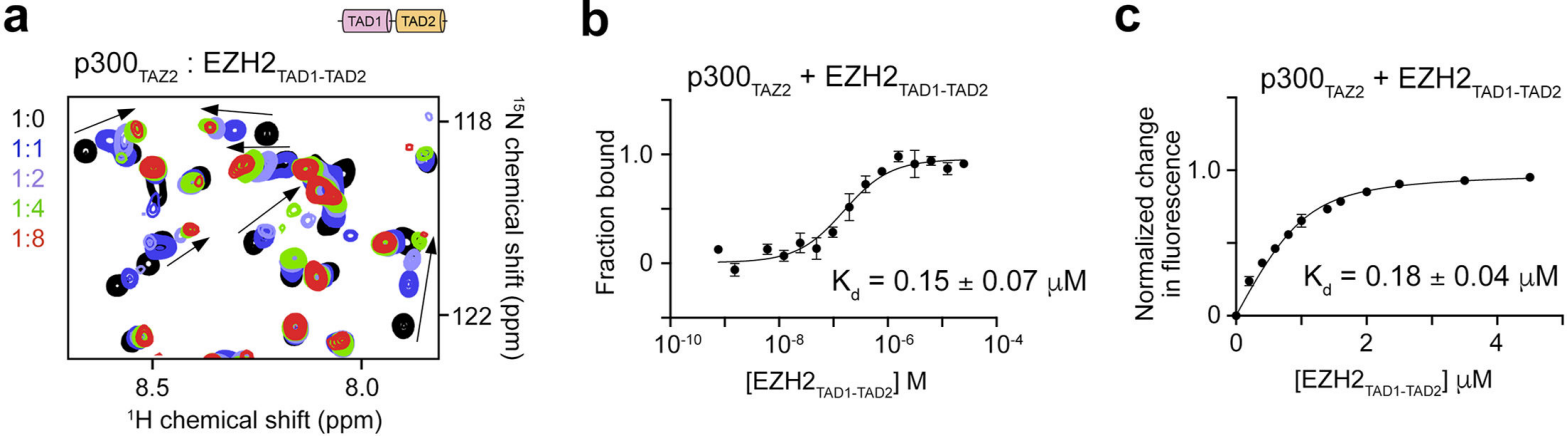
p300_TAZ2_ binds stronger to the linked EZH2_TAD1-TAD2_ . (**a**) Overlaid ^1^H,^15^N HSQC spectra of ^15^N-labeled p300_TAZ2_ collected in the absence (black) and presence of EZH2_TAD1-TAD2_ (aa 142–185 of EZH2). Spectra are color-coded according to the protein:peptide molar ratio. See also Supplementary Figure S1. (**b**) Normalized binding curve used to determine apparent binding affinity of p300_TAZ2_-His to EZH2_TAD1-TAD2_ by MST. Data and point errors are the average of four independent measurements ± SEM. The apparent K_d_ value is the average of four independent measurements ± SD. (**c**) Normalized binding curve used to determine apparent K_d_ for the interaction of p300_TAZ2_-His with EZH2_TAD1-TAD2_ by tryptophan fluorescence. Data and point errors are the average of three independent measurements ± SD. The apparent K_d_ value is the average of three independent measurements ± SD.

We tested the binding of p300_TAZ2_ to a longer EZH2 peptide, containing both EZH2_TAD1_ and EZH2_TAD2_ (EZH2_TAD1-TAD2_) by NMR. Titration of the dual EZH2_TAD1-TAD2_ led to large CSPs in ^1^H,^15^N HSQC spectra of p300_TAZ2_ (Figure 4a). Binding affinity of p300_TAZ2_ to EZH2_TAD1-TAD2_, measured by MST (K_d_ of 0.15 µM) and tryptophan fluorescence (K_d_ of 0.18 µM), revealed a ~two-fold increase in binding affinity compared with the binding affinity of p300_TAZ2_ to EZH2_TAD2_ and a ~three-fold increase compared with the binding affinity to EZH2_TAD1_ (Figure 4b and c). Such an increase is likely due to an increase in apparent concentration of the binding sites in the linked EZH2_TAD_s.

**Figure 5:**
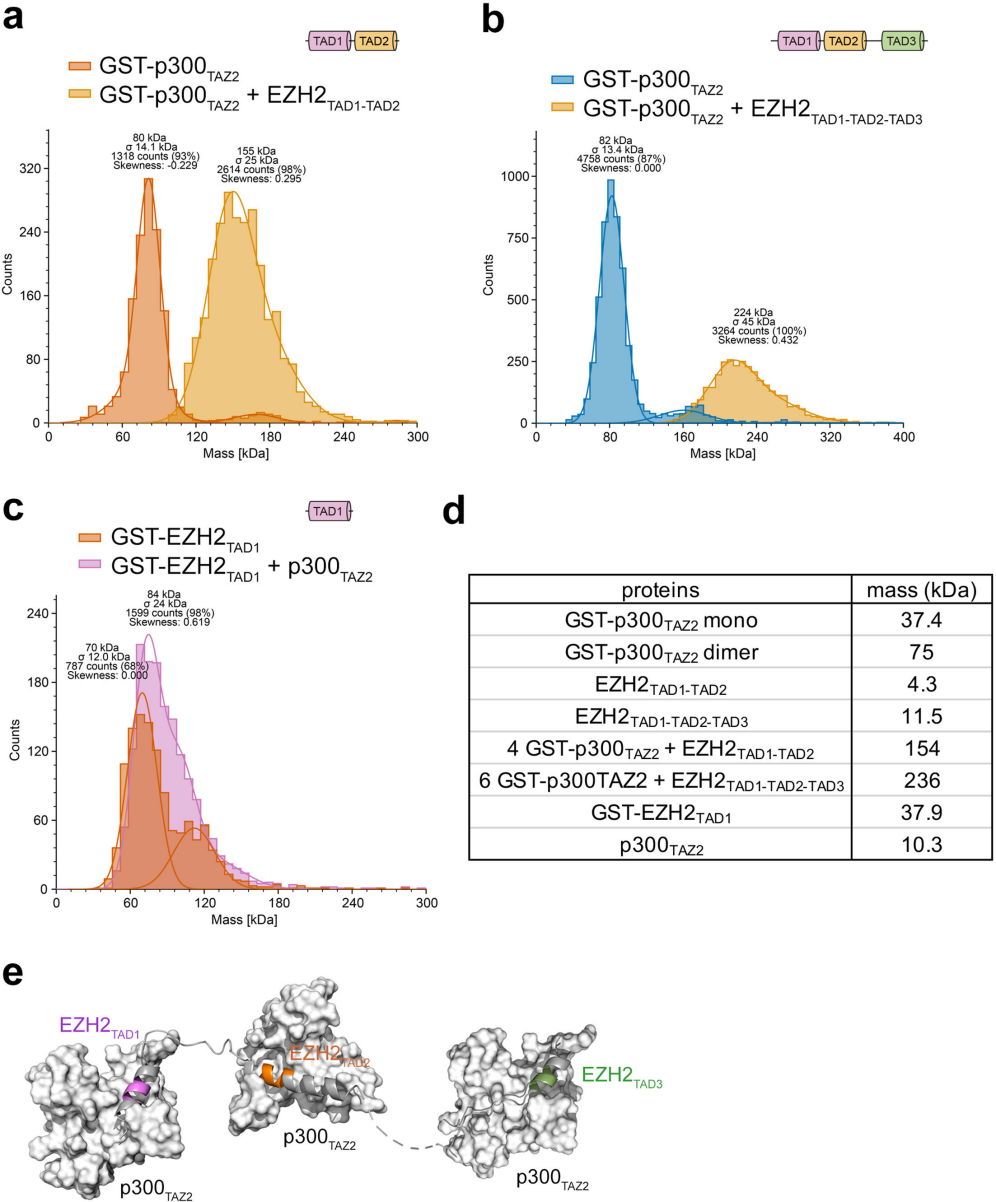
p300_TAZ2_ and EZH2_TAD_ form oligomeric complexes. (**a-c**) MP histograms of GST-p300_TAZ2_ in the absence or presence of (**a**) EZH2_TAD1-TAD2_ or (**b**) EZH2_TAD1-TAD2-TAD3_, or (**c**) GST-EZH2_TAD1_ in the absence or presence of p300_TAZ2_. (**d**) Table of calculated molecular masses of the complexes relevant to MP data. (**e**) A model of the concurrent association of p300_TAZ2_ with each of EZH2_TAD_s.

The finding that each EZH2_TAD_ can be bound by a separate p300_TAZ2_ was substantiated in mass photometry (MP) assays (Figure 5). The MP measurements showed that in the apo-state, GST-p300_TAZ2_ exists in a dimeric form (MP peak of ~80 kDa) due to the ability of GST to dimerize (Figure 5a). The addition of the dual EZH2_TAD1-TAD2_ resulted in the formation of the complex of molecular mass corresponding to four molecules of GST-p300_TAZ2_ and one molecule of EZH2_TAD1-TAD2_ (MP peak of ~155 kDa). These data indicated that one dimer of GST-p300_TAZ2_ interacts with EZH2_TAD1_, whereas another dimer of GST-p300_TAZ2_ interacts with EZH2_TAD2_. Furthermore, the addition of the triple EZH2_TAD1-TAD2-TAD3_ led to the formation of the complex of molecular mass corresponding to six molecules of GST-p300_TAZ2_ and one molecule of EZH2_TAD1-TAD2-TAD3_ (MP peak of ~224 kDa) (Figure 5b). Collectively, MP data revealed that each EZH2_TAD_ can be bound by a separate p300_TAZ2_ (Figure 5e). This was further corroborated by MP measurements using GST-EZH2_TAD1_ which formed a dimer in the apo-state again due to the ability of GST to dimerize (MP peak of ~70 kDa). The addition of p300_TAZ2_ to GST-EZH2_TAD1_ led to a ~14 kDa shift of the MP peak, suggesting the presence of two complexes, dimeric GST-EZH2_TAD1_ bound by one molecule of p300_TAZ2_ and by two molecules of p300_TAZ2_.

### EZH2 stimulates p300 HAT activity

**Figure 6:**
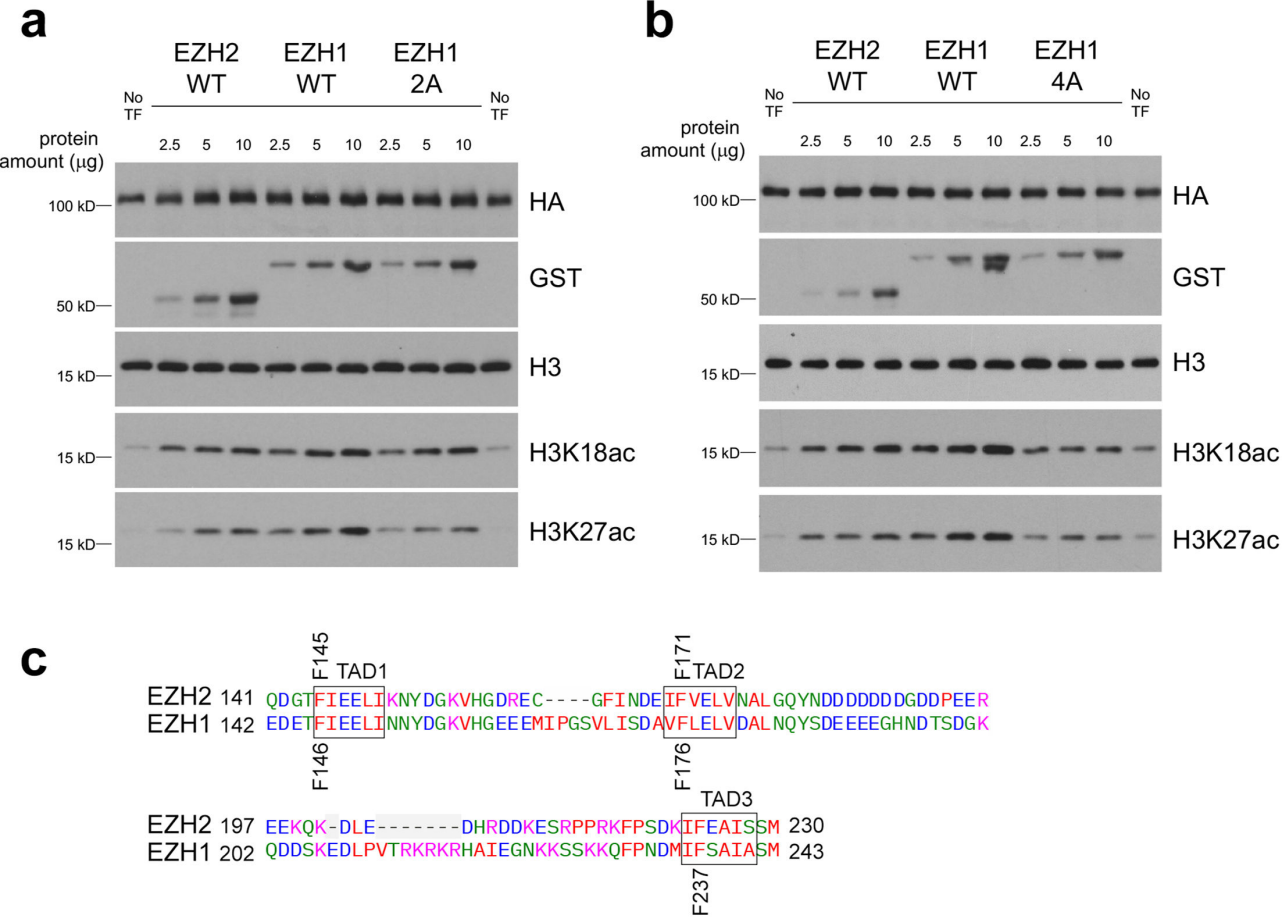
TADs of EZH2 and EZH1 stimulate p300 HAT activity. (**a, b**) Western blot analysis of HAT assays of the FLAG-HA-tagged p300 catalytic core (aa 1035–1830 of p300) in the absence and presence of increasing amounts of GST-tagged EZH2_TAD1-TAD2-TAD3_ (aa 119–267, EZH2 WT), GST-tagged EZH1_TAD1-TAD2-TAD3_ (aa 120–280, EZH1 WT), GST-tagged F176A/F237A mutant of EZH1_TAD1-TAD2-TAD3_ (aa 120–280, EZH1 2A) or GST-tagged F146A/F176A/F237A/Y257A mutant of EZH1_TAD1-TAD2-TAD3_ (aa 120–280, EZH1 4A) on the recombinant nucleosome. Blots were probed with H3K18ac and H3K27ac antibodies. Total H3 was used as loading control. (**c**) Sequence alignment of the EZH2 and EZH1 TADs. Mutated residues are labeled.

We have previously shown that p300_TAZ2_ mediates acetyltransferase function of p300 [41]. To explore whether binding of EZH2_TAD_s affects the catalytic activity of the p300 HAT domain, we purified FLAG-HA-tagged p300 catalytic core (aa 1035–1830 of p300) from 293 T cells and tested it in HAT assays using the recombinant nucleosome as a substrate (Figure 6). As shown in Figure 6a and b, the FLAG-HA-tagged p300 catalytic core alone inefficiently acetylates histone H3K18 and H3K27. The addition of GST-tagged EZH2_TAD1-TAD2-TAD3_ (aa 119–267, EZH2 WT) or homologous GST-tagged EZH1_TAD1-TAD2-TAD3_ (aa 120–280, EZH1 WT) to the reactions led to a dose-dependent increase in H3K18 and H3K27 acetylation. The EZH1-dependent stimulation of the p300 enzymatic activity was decreased when F176 and F237 in TAD2 and TAD3 of EZH1 (EZH1 2A in Figure 6a) were mutated to alanine and was further decreased when additionally F146 in TAD1 and Y257 of EZH1 (EZH1 4A in Figure 6b) were substituted to alanine. We note that F145A and F171A mutations in EZH2 (equivalent to F146A and F176A in EZH1) were previously shown to disrupt binding to p300 in pull-down assays [20]. Collectively, these data indicated that binding of EZH1/2_TAD_s to p300_TAZ2_ stimulates the acetyltransferase function of p300.

### p300 and EZH2 co-occupy non-canonical genomic sites of EZH2 in prostate cancer cells

**Figure 7:**
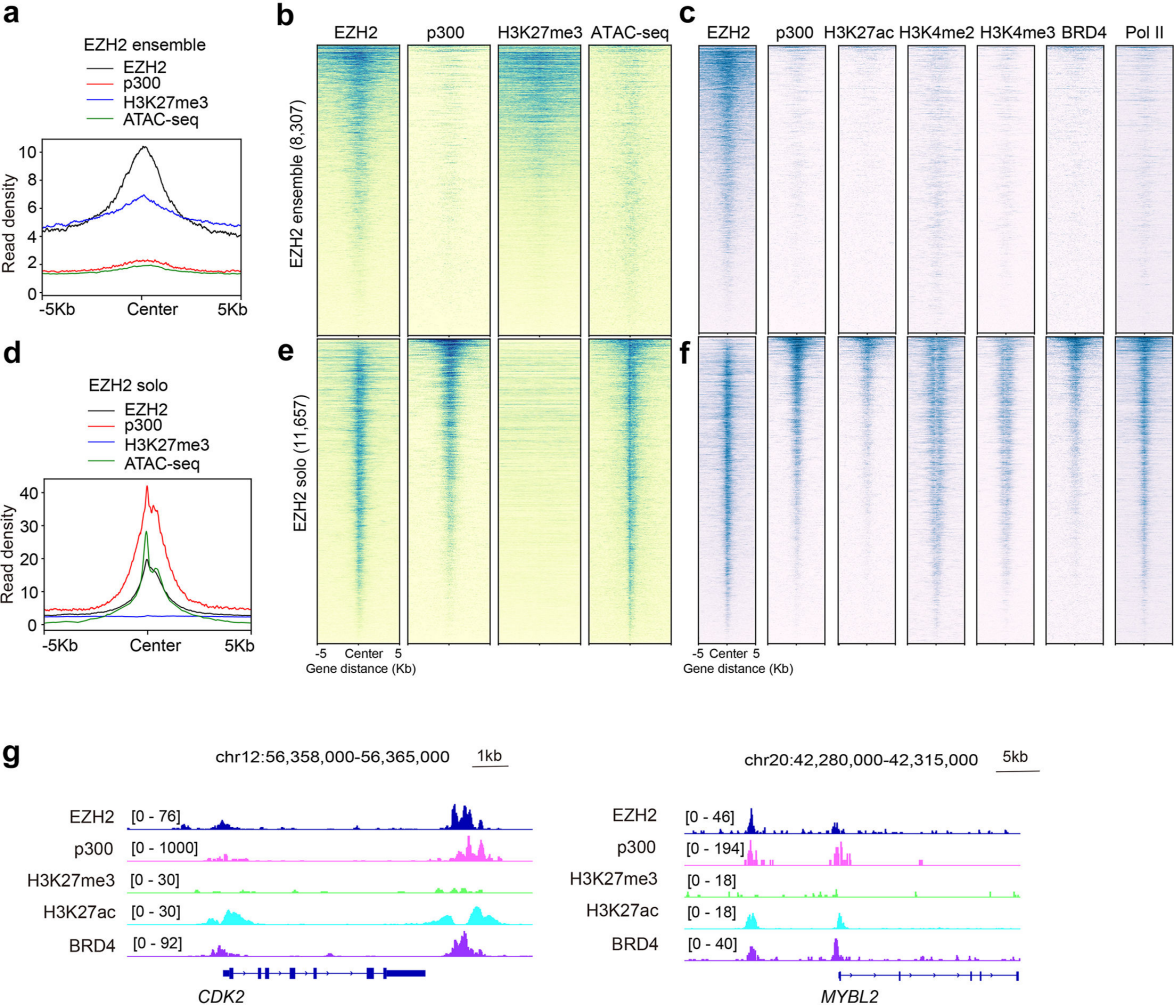
EZH2 and p300 co-localize at EZH2’s non-canonical genomic sites. (**a-c)** Averaged intensities (**a**) and heatmap (**b**) of EZH2 or H3K27me3 CUT&RUN, p300 CUT&Tag and ATAC-seq signals, ±5 kb from the centers of canonical EZH2 peaks (i.e. EZH2-ensemble peaks), which show the EZH2 and H3K27me3 co-binding but low levels of p300, gene-active histone marks, BRD4 and RNA Pol II (**c**). (**d-f**) Averaged intensities (**d**) and heatmap (**e**) of EZH2 or H3K27me3 CUT&RUN, p300 CUT&Tag and ATAC-seq signals, ±5 kb from the centers of non-canonical EZH2 peaks (i.e. EZH2-solo peaks), with the EZH2 binding sites lacking H3K27me3 but showing high levels of p300, gene-active histone marks, BRD4 and RNA Pol II (**f**). (**g**) Integrative genomics viewer plots of enrichment of EZH2 and p300, as well as the transcriptional coactivator BRD4 and H3K27ac at the two known oncogenes, *CDK2* and *MYBL2* (also known as *B-MYB*).

To determine whether the genomic binding sites of p300 and EZH2 correlate in the cellular context, we performed cleavage under targets and tagmentation (CUT&Tag) experiments for p300 in 22Rv1 cells that are commonly used as a prostate cancer cell model. For comparative genomic profiling analyses, we used the publicly available datasets, such as assay for transposase-accessible chromatin using sequencing (ATAC-seq), chromatin immunoprecipitation followed by sequencing (ChIP-seq), and cleavage under targets and release using nuclease (CUT&RUN) in the same 22Rv1 cells. Using the EZH2 and H3K27me3 peaks as input, an unsupervised clustering analysis was performed to define categories of genomic sites showing differential binding patterns. First, we observed a co-binding pattern for EZH2 and H3K27me3, a repressive PTM associated with the catalytic activity of the canonical EZH2:PRC2 complex, at a set of genomic sites (8307 peaks), termed the EZH2 ensemble sites (Figure 7a and b). As expected, these canonical EZH2:PRC2 target sites were lacking p300 and other markers of transcriptionally active chromatin, including H3K27ac, H3K4me2, H3K4me3, the transcriptional coactivator BRD4, and RNA polymerase II (Pol II) (Figure 7a–c). Surprisingly, we also identified a large set of the EZH2 peaks (termed EZH2-solo) lacking H3K27me3 (11,657 peaks) (Figure 7d–f). In contrast to the canonical EZH2:PRC2 binding sites, EZH2-solo binding sites were characterized by substantial p300 co-occupancy and high levels of H3K27ac (an active histone mark deposited by p300), H3K4me2, H3K4me3, BRD4, and RNA Pol II. Co-localization of EZH2, p300, and BRD4 and high level of H3K27ac but the lack of H3K27me3 were obvious at *CDK2* and *MYBL2*, two well-recognized oncogenes (Figure 7g). Together, these results indicated that EZH2 and p300 co-localize at a large set of genes in 22Rv1 prostate cancer cells.

**Figure 8:**
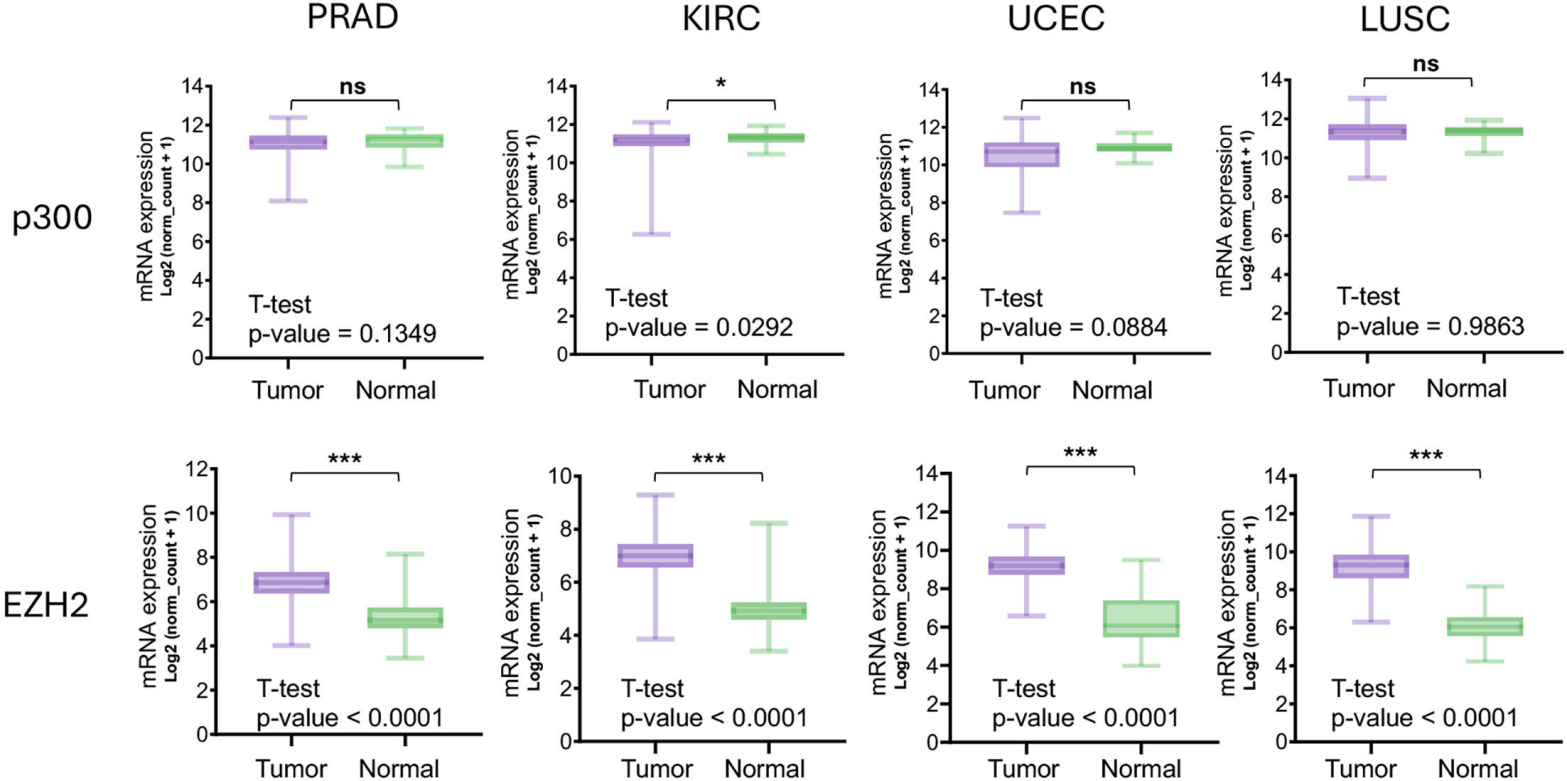
Differential expression of p300 and EZH2 between normal and tumor tissues across four cancer types. Normalized mRNA expression levels of p300 and EZH2 were compared between normal and tumor tissues in PRAD, KIRC, UCEC, and LUSC datasets from TCGA, obtained via UCSC Xena cancer genomics database. Statistical significance was assessed using T-tests, with significant differences indicated by *P* values (*P*<0.05). Asterisks represent statistical significance.

An analysis of mRNA expression levels of EZH2 and p300 in prostate adenocarcinoma (PRAD), kidney renal clear cell carcinoma (KIRC), uterine corpus endometrial carcinoma (UCEC), and lung squamous cell carcinoma (LUSC) from The Cancer Genome Atlas (TCGA) showed that EZH2 was consistently overexpressed in tumor tissues compared with normal tissues (Figure 8). The differences were statistically significant (*P*<0.05) in agreement with previous reports demonstrating the canonical oncogenic activity of EZH2 [42]. PRAD patients with high EZH2 expression had a poor prognosis, with significantly shorter progression-free survival compared with patients with low EZH2 expression (Supplementary Figure S2). In contrast, no significant differences in the expression level of p300 were detected between normal tissues and these four cancer types (Figure 8). Furthermore, correlation analysis across tumor samples revealed no significant association between p300 and EZH2 expression, indicating that their transcriptional regulation occurs independently (Supplementary Figure S3).

### Concluding remarks

Both loss-of-function and gain-of-function alterations of EZH2 have been identified in various cancer types. Many of these alterations reflect the cell type-specific tumor-suppressive or oncogenic activities of PRC2, which usually rely on H3K27me3-mediated gene repression [1,43]. In contrast, emerging evidence suggests that EZH2 functions beyond the PRC2 context and plays a role in activating gene expression. In the present study, we show that in 22Rv1 prostate cancer cells, EZH2 occupies a large set of gene loci lacking H3K27me3. These non-canonical genomic sites of EZH2 are instead co-bound by p300, RNA Pol II, and BRD4 and are rich in histone marks associated with transcriptional activation. Our findings suggest a mechanism for such a high degree of genetic correlation through the direct binding of p300_TAZ2_ to EZH2_TAD_s and the subsequent stimulation of acetyltransferase activity of p300. Both the close proximity of EZH2_TAD_s that increases the apparent concentration of the ligand and the ability of three p300_TAZ2_ to simultaneously interact with all three EZH2_TAD_s could play a role in bridging EZH2 and p300. As p300_TAZ2_ has been shown to bind DNA, in future work, it will be interesting to assess the contribution of this binding to the EZH2-p300 complex formation. Elucidation of the molecular basis for the EZH2-p300-chromatin assembly is also essential for our understanding of the etiology of cancers associated with non-canonical PRC2-independent function of EZH2.

## Methods

### Protein purification

Human p300_TAZ2_ [aa 1722–1812, pGEX-4T-1 vector with an additional tobacco etch virus (TEV) cleavage site] with a tryptophan substituted at A1723 and p300_TAZ2_-6×His constructs were purified essentially as in Becht et al. [37]. Briefly, both ^15^N-labeled and unlabeled constructs were expressed in *Escherichia coli* Rosetta-2 (DE3) pLysS cells grown in either M9 minimal media or TB, both supplemented with 50–150 μM ZnCl_2_. After induction with isopropyl b-D-1-thiogalactopyranoside (IPTG), harvesting, and lysis in buffer [50 mM Tris pH 7.0, 1 M NaCl, 5 mM dithiothreitol (DTT), 1 mM phenylmethanesulfonylfluoride (PMSF) and DNase] by sonication, the proteins were purified on glutathione agarose beads (Pierce) and eluted with buffer containing reduced L-glutathione (Fisher). The GST tag was either cleaved with TEV protease overnight at 22°C or kept intact during subsequent purification steps. Both GST- and His-tagged proteins, as well as untagged p300_TAZ2_, were further purified by cation exchange (HiTrap SP HP, Cytiva) or heparin affinity chromatography (HiTrap Heparin HP, Cytiva).

Human EZH2_TAD1-TAD2-TAD3_ (aa 142–231) construct with a C-terminal 6×His tag was cloned into a pGEX-6P-1 vector (GenScript). The EZH2_TAD1_ (aa 142–160) construct was generated by deletion using Q5 Hot Start High-Fidelity kit (NEB) and confirmed by DNA sequencing. Both constructs were expressed in *E. coli* Rosetta-2 (DE3) pLysS cells grown in LB media and induced at an OD_600_ of 0.6–0.8 with 1 mM IPTG for 16 h at 16°C. Cells were harvested by centrifugation, suspended in buffer (50 mM Tris pH 7.5, 500 mM NaCl, 1% CHAPS, 2 mM DTT, 1 mM PMSF, Roche cOmplete Protease Inhibitor Cocktail, and DNase), and lysed by sonication. The doubly-GST- and -His-tagged EZH2_TAD_ constructs were purified on glutathione agarose beads (Pierce), eluted with buffer containing 50 mM reduced L-glutathione, then exchanged into relevant experimental buffers using 10 kDa MWCO centrifugal concentrators (Millipore). For MP, EZH2_TAD_ constructs were either cleaved with PreScission protease or left with GST tag intact, followed by purification on Ni-NTA beads (HisPur, Thermo Sci.), washed with buffer (50 mM Tris pH 7.5, 500 mM NaCl, 30 mM imidazole, 1% CHAPS, 2 mM DTT), and eluted with buffer containing 300 mM imidazole. When necessary, EZH2_TAD_ constructs were further purified using a Superdex 200 Increase 10/300 GL column (Cytiva) with monitoring at 205 and 280 nm.

EZH2_TAD1_ (aa 142–154 of EZH2), EZH2_TAD2_ (aa 167–185 of EZH2), EZH2_TAD3_ (aa 220–231 of EZH2), and EZH2_TAD1-TAD2_ (aa 142–185 of EZH2) peptides were synthesized by SynPeptide Co. Ltd.

### NMR experiments

NMR experiments were performed at 298 K on a Varian INOVA 600 MHz spectrometer equipped with a cryoprobe. The ^1^H,^15^N HSQC spectra of 50–100 μM uniformly ^15^N-labeled proteins were collected in the presence of an increasing amount of peptide (SynPeptide) in buffer containing 50 mM MES pH 6.5, 150 mM NaCl, 5 mM DTT. NMR data were processed and analyzed with NMRPipe and NMRDraw as previously described [44]. Normalized chemical shift changes were calculated using the following equation:


$$\Delta \delta =\sqrt{{\left(\Delta \delta H\right)}^{2}+{\left(0.14*\Delta \delta N\right)}^{2}}$$

where Δδ is the change in chemical shift in parts per million (ppm). CSP graphs were analyzed with CcpNmr Analysis v3.2 and generated in GraphPad PRISM.

### Tryptophan fluorescence

Fluorescence spectra were collected at 25°C on a Fluoromax spectrofluorometer (HORIBA). Sample contained 1 μM protein in a buffer containing 25 mM Tris pH 7.5, 150 mM NaCl, and 2.5 mM DTT. p300_TAZ2_ in the absence and presence of increasing concentrations of the EZH2_TAD1-TAD2_ peptide was excited at 295 nm, and emission spectra were recorded between 320 and 360 nm with a 0.5-nm step size and a 0.5-s integration time. The apparent [45] K_d_ estimated for EZH2_TAD1-TAD2_ peptide was determined by a nonlinear least-squares analysis using a 1:1 stoichiometry and the following equation:


$$∆I={∆I}_{max}\left(\left(\left[L\right]\right.+\left[P\right]+K_{d})-\sqrt{{\left(\left[L\right]+\left[P\right]+K_{d}\right)}^{2}-4\left[P\right]\left[L\right])}\right)/2\left[P\right]$$

where [L] is concentration of the peptide, [P] is concentration of the protein, ΔI is the observed change of signal intensity, and ΔI_max_ is the difference in signal intensity of the free and bound states of the protein. The K_d_ value was averaged over three independent experiments with error calculated as the standard deviation between the runs.

### MST

MST experiments were carried out on a Monolith NT.115 instrument (NanoTemper). All experiments were performed using SEC-purified p300_TAZ2_-6×His protein in 25 mM Tris pH 7.5 buffer, 150 mM NaCl, and 2.5 mM DTT. p300_TAZ2_-6×His was labeled using a His-Tag Labeling Kit RED-tris-NTA (2nd Generation, NanoTemper) and kept constant at 10 nM. Dissociation constants were determined using a direct binding assay in which peptide was varied in concentration by serial dilution of discrete samples. The measurements were performed at 50% LED and medium MST power with 3-s pre-laser time, 20-s laser on-time, and 1-s off-time. The K_d_ values, including apparent K_d_ estimated for EZH2_TAD1-TAD2_ peptide, were calculated using MO Affinity Analysis software (NanoTemper) using a 1:1 stoichiometry and averaged over three or four separate experiments with error reported as SD. Plots were generated in GraphPad PRISM.

### Mass Photometry

MP experiments were conducted using a Refeyn TwoMP mass photometer (Refeyn Ltd, Oxford, U.K) to monitor the formation of oligomeric states involving p300_TAZ2_ and TADs of EZH2. To overcome the minimum size limit, a GST-fusion construct was used for one of the components in each sample. In summary, samples contained either 20 nM GST-p300_TAZ2_ in the absence and presence of EZH2_TAD1-TAD2_ (5 μM) or EZH2_TAD1-TAD2-TAD3_ (1 μM) or 20 nM GST-EZH2_TAD1_ with or without p300_TAZ2_ (0.1 μM). Ten microliter of each sample was loaded into the sample wells of the silicon cassettes assembled onto MassGlass UC coverslips (Refeyn, Ltd.). Measurements were performed at room temperature in buffer (50  mM Tris pH 7.5, 150  mM NaCl, 5  mM DTT). β-amylase was used as calibration standard (56  kDa, 112  kDa, and 224  kDa). After the focus was set and locked, movies were captured for 60  s (2800 frames) using AcquireMP software (Refeyn Ltd). Data were processed using DiscoverMP (Refeyn Ltd).

### Histone acetyltransferase assay

For HAT assays, FLAG-HA-tagged p300 core (aa 1035–1830 of p300) in a pCDH vector was expressed in 293 T cells for two days. Cells were lysed in lysis buffer (50 mM Tris-HCl pH 7.5, 300 mM NaCl, 1.5 mM MgCl_2_, 1 mM EDTA, 1% Triton100, 10% glycerol, 1 mM PMSF, and protease inhibitors) and sonicated. Cell lysates were incubated with anti-Flag M2 beads (Sigma) at 4° for 4 h. The beads were then washed three times with buffer (50 mM Tris-HCl pH 7.5, 500 mM NaCl, 1.5 mM MgCl_2_, 0.2 mM EDTA, 0.1% NP40, 10% glycerol, and 1 mM PMSF), and the protein was eluted with elution buffer (50 mM Tris-HCl pH 7.5, 300 mM NaCl, 1 mM DTT, 10% glycerol, and 0.4 mg/ml Flag peptide).

GST-tagged EZH2_TAD_s (aa 119–267), EZH1_TAD_s (aa 120–280), F176A/F237A EZH1_TAD_s (EZH1 2A), and F146A/F176A/F237A/Y257A EZH1_TAD_s (EZH1 4A) constructs in the pGEX-6p-1 vector were transformed into RosettaTM2 (DE3) pLysS competent cells (Novagen). Protein production was induced by incubating with 0.4 mM IPTG at 16°C for 20 h in LB. Cell pellets were resuspended in lysis buffer (50 mM Tris-HCl pH 8.0, 300 mM NaCl, 1 mM PMSF, and 1 × cOmpleteTM EDTA-free Protease Inhibitor Cocktail) and lysed by sonication. Lysates were centrifuged at 17,000 ***g*** for 15 min, and supernatants were incubated with Glutathione Sepharose® 4B beads (Sigma) at 4°C for 2 h. Beads were washed with lysis buffer twice, and proteins were eluted with elution buffer (50 mM Tris-HCl pH 8.0, 150 mM NaCl, 10% glycerol, and 15 mg/ml GSH).

Purified FLAG-HA-tagged p300 catalytic core fragment (1 μg) was incubated with increasing amounts of GST-tagged EZH2_TAD_s and GST-tagged EZH1_TAD_s (2.5, 5, or 10 μg) and the nucleosome (1 μg) in HAT reaction buffer (50 mM Tris-HCl pH 8.0, 100 mM NaCl, 10% glycerol, 1 mM DTT, 100 mM acetyl-CoA, and 1× cOmplete EDTA-free Protease Inhibitor Cocktail) at 30°C for 15 min. Reactions were stopped by boiling at 95°C for 5 min in 2×SDS loading buffer, and samples were analyzed by SDS-PAGE and Western blot. The following antibodies were used: HA (CST, 3724, 1:2000), GST (Santa Cruz, sc-459, 1:2000), H3 (Abcam, ab1791, 1:80,000), H3K18ac (ActiveMotif, 39755, 1:1000), and H3K27ac (Abcam, ab4729, 1:1000).

### Genomic profiling and data analysis

The 22Rv1 cells, a classic prostate cancer cell model, were obtained from American Tissue Culture Collection (ATCC, CRL-2505) and cultured according to the vendor-provided protocol. CUT&Tag was performed using the commercial EpiCypher CUTANA kit and following the manufacturer’s detailed protocols. Briefly, 100,000 human tumor cells were used in each assay followed by sample preparation, multiplex library preparation, and deep sequencing (Illumina NextSeq 1000). The primary antibodies of p300 (Cell Signaling cat# 54,062S) were used at a 1:100 concentration. The public genomic datasets of 22Rv1 cells included those of NCBI Gene Expression Omnibus (GEO) database under the accession number GSE205107 (EZH2 and H3K27me3 CUT&RUN data of 22Rv1 cells), GSE99378 (ATAC-seq data of 22Rv1 cells), GSM2827408 and GSE85558 (ChIP-seq for histone mark in 22Rv1 cells), and GSE94013 (ChIP-seq for BRD4 in 22Rv1 cells).

Genomic data were analyzed as before [20]. Briefly, fastq files (trim_galore) were mapped to the reference genome (hg38) using bowtie2 (v.2.4.4). The non-primary alignment and PCR duplicates were removed from aligned data using Samtools (v.1.10) (-q 30 F 1804 f 2 for unique mapping reads), the Picard ‘MarkDuplicates’ function (v.2.18.2), and bedtools (v.2.30.0) (‘intersect’ function used to exclude genome blacklist regions), respectively. Peak calling was performed using MACS2 (v.2.2.6) (macs2 callpeak -f BAMPE -g hs/mm –keep-dup 1 --cutoff-analysis -q 0.05). For spike-in samples, fastq files were mapped to the reference genome using STAR (v.2.7.11a). Then, non-primary alignment and PCR duplicate alignments were removed by Samtools (v.1.10) (-F 256) and Picard ‘MarkDuplicates’ function (v.2.18.2). Deeptools (v3.3.0) was used to generate bigwig files. Genomic binding profiles were generated by using the deepTools ‘bam-Compare’ functions. An unsupervised clustering function of deepTools was used to analyze the EZH2 and H3K27me3 peaks and define categories of genomic sites showing different binding patterns, such as the EZH2 ensemble sites and the EZH2 solo sites.

### Gene expression and correlation analysis in tumor samples

Gene expression data for p300 and EZH2 were retrieved from cBioPortal for Cancer Genomics (https://www.cbioportal.org/) and UCSC Xena (https://xena.ucsc.edu/). Datasets from TCGA for PRAD, KIRC, UCEC, and LUSC were used in analysis. RNA-Seq expression data, normalized using RSEM and batch-corrected, were analyzed across tumor samples for each cancer type. Correlation analysis between p300 and EZH2 mRNA expression was performed using Pearson or Spearman correlation coefficients, with statistical significance set at *P*<0.05. For the PRAD dataset, samples were stratified into quartiles based on EZH2 expression levels, and patient progression-free survival was compared between the highest (top 25%) and lowest (bottom 25%) quartiles.

## Supplementary material

Online supplementary figures

## Data Availability

All relevant data supporting the key findings of this study are available within the article and the Supplementary Information file.

## Open Access

This article has been published open access under our Subscribe to Open programme, made possible through the support of our subscribing institutions, learn more here: https://portlandpress.com/pages/open_access_options_and_prices#conditional

## Abbreviations

ATAC-seq: assay for transposase-accessible chromatin using sequencing
CUT&RUN: cleavage under targets and release using nuclease
CUT&Tag: cleavage under targets and tagmentation
ChIP-seq: chromatin immunoprecipitation followed by sequencing
DTT: dithiothreitol
EZH2: enhancer of zeste homologue 2
HAT: histone acetyltransferase
IPTG: isopropyl b-D-1-thiogalactopyranoside
KIRC: kidney renal clear cell carcinoma
LUSC: lung squamous cell carcinoma
MP: mass photometry
PMSF: phenylmethanesulfonylfluoride
PRAD: prostate adenocarcinoma
PRC2: *polycomb* repressive complex 2
TADs: transactivation domains
TCGA: The Cancer Genome Atlas
TEV: tobacco etch virus
UCEC: uterine corpus endometrial carcinoma
