# Supplementary material for: Multiple transactivation domains of EZH2 bind to the TAZ2 domain of p300 and stimulate the acetyltransferase function of p300
Source: Biochem J. 2025 Jul 2;482(13):955–68. doi: 10.1042/BCJ20253037 (PMC12312386; doi:10.1042/BCJ20253037)
Supplement: Online supplementary figures [file bcj-482-13-BCJ20253037-s001.pdf]

Supplementary Information

Multiple transactivation domains of EZH2 bind to the TAZ2 domain of p300 and stimulate acetyltransferase function of p300

Dustin C. Becht, et al.,

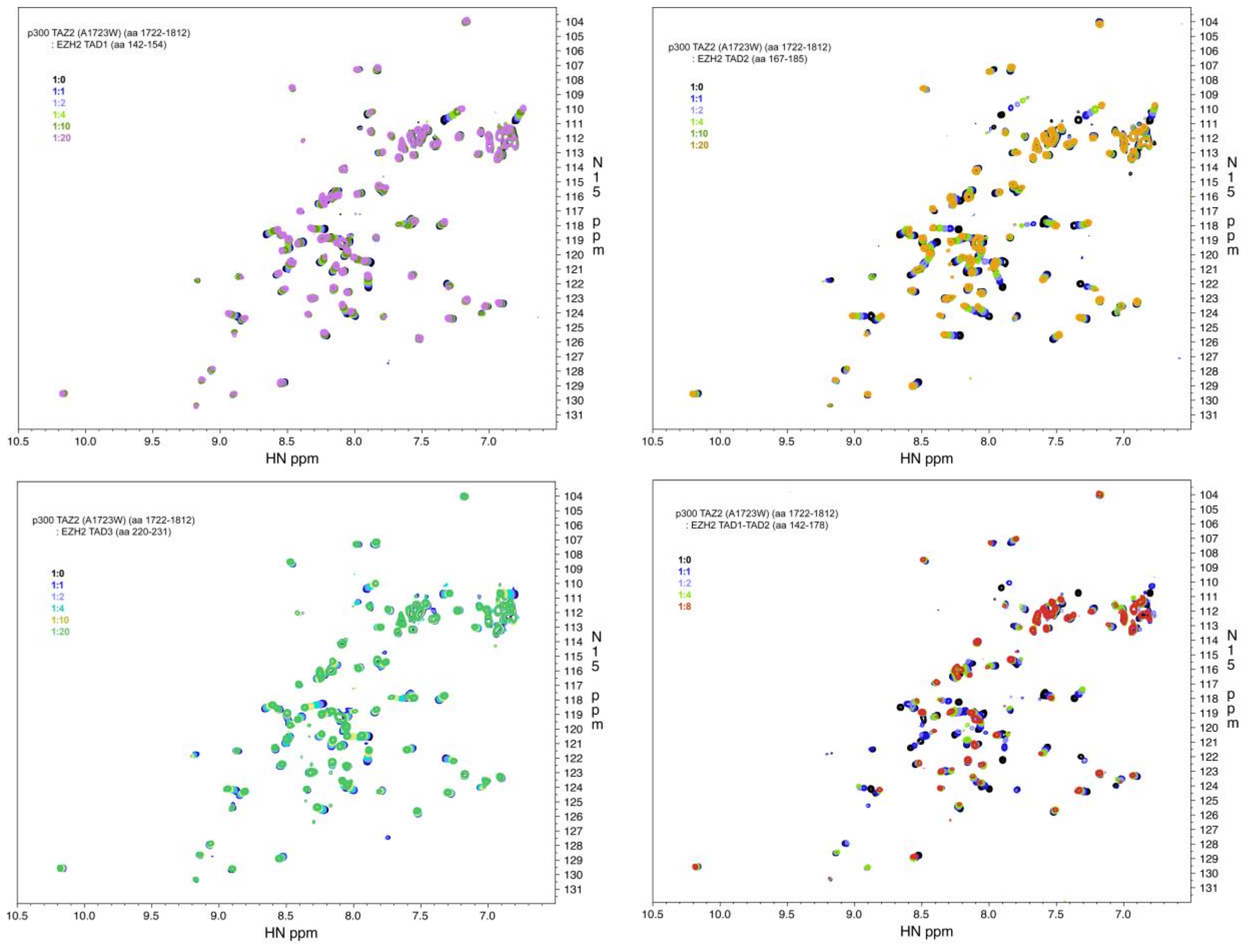

Supplementary Figure 1. Superimposed ^{1}H,^{15}N HSQC spectra of p300$_{TAZ2}$ (A1723W) recorded in the presence of increasing amounts of the indicated EZH2 peptides. The spectra are color coded according to the protein:peptide molar ratio. Related to Figures 2 and 4.

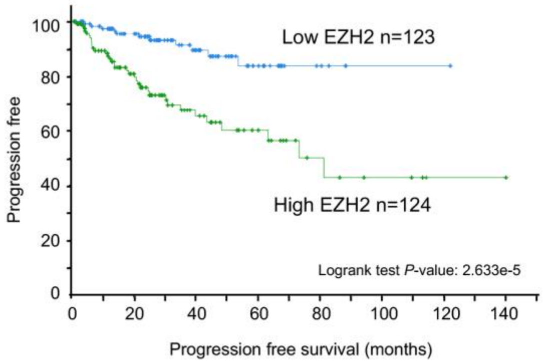

Supplementary Figure 2. Kaplan-Meier survival curves illustrate Progression-Free Survival (PFS) in patients stratified by EZH2 expression levels. Tumor samples from the PRAD dataset (TCGA) were stratified into quartiles based on EZH2 expression levels. mRNA expression was compared between the highest (top 25%) and lowest (bottom 25%) gene expression quartiles. Statistical significance was determined using appropriate statistical tests, with significant differences indicated ($p < 0.05$). Related to Figure 8.

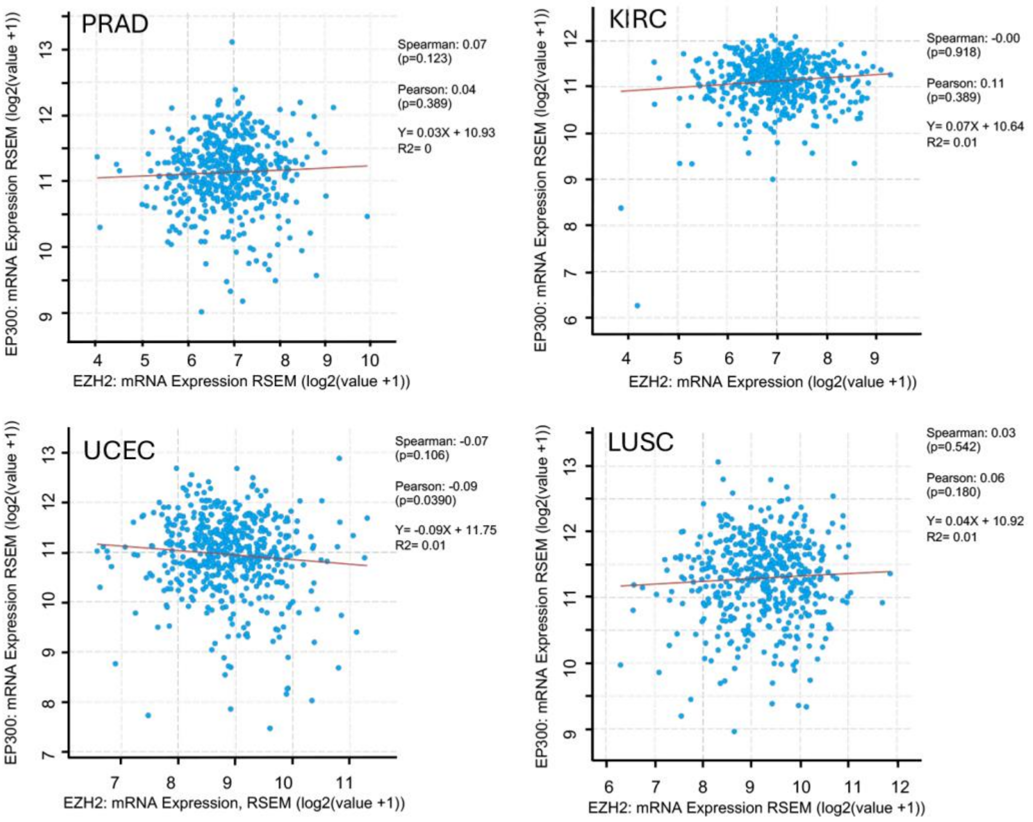

Supplementary Figure 3. Correlation analysis between p300 and EZH2 expression in tumor samples. Correlation analysis of p300 (EP300) and EZH2 expression was conducted across tumor samples using data from cBioPortal. Pearson or Spearman correlation coefficients were calculated to evaluate mRNA expression relationships in tumors. Related to Figure 8.
